# G-CSF protects motoneurons against axotomy-induced apoptotic death in neonatal mice

**DOI:** 10.1186/1471-2202-11-25

**Published:** 2010-02-23

**Authors:** Alexandre Henriques, Claudia Pitzer, Luc Dupuis, Armin Schneider

**Affiliations:** 1 SYGNIS Bioscience, Im Neuenheimer Feld 515, 69120 Heidelberg, Germany; 2 University of Strasbourg, Faculty of Medicine, UMRS692, Strasbourg, France; 3 INSERM U692, Strasbourg, France

## Abstract

**Background:**

Granulocyte colony stimulating factor (G-CSF) is a growth factor essential for generation of neutrophilic granulocytes. Apart from this hematopoietic function, we have recently uncovered potent neuroprotective and regenerative properties of G-CSF in the central nervous system (CNS). The G-CSF receptor and G-CSF itself are expressed in α motoneurons, G-CSF protects motoneurons, and improves outcome in the SOD1(G93A) transgenic mouse model for amyotrophic lateral sclerosis (ALS). In vitro, G-CSF acts anti-apoptotically on motoneuronal cells. Due to the pleiotrophic effects of G-CSF and the complexity of the SOD1 transgenic ALS models it was however not possible to clearly distinguish between directly mediated anti-apoptotic and indirectly protective effects on motoneurons. Here we studied whether G-CSF is able to protect motoneurons from purely apoptotic cell death induced by a monocausal paradigm, neonatal sciatic nerve axotomy.

**Results:**

We performed sciatic nerve axotomy in neonatal mice overexpressing G-CSF in the CNS and found that G-CSF transgenic mice displayed significantly higher numbers of surviving lumbar motoneurons 4 days following axotomy than their littermate controls. Also, surviving motoneurons in G-CSF overexpressing animals were larger, suggesting additional trophic effects of this growth factor.

**Conclusions:**

In this model of pure apoptotic cell death the protective effects of G-CSF indicate direct actions of G-CSF on motoneurons in vivo. This shows that G-CSF exerts potent anti-apoptotic activities towards motoneurons in vivo and suggests that the protection offered by G-CSF in ALS mouse models is due to its direct neuroprotective activity.

## Background

Granulocyte-colony-stimulating factor is a cytokine that stimulates the proliferation and the differentiation of myeloid precursors [1] and has been in clinical use for more than 10 years in indications related to counteracting chemotherapy-induced neutropenia or for bone-marrow transplantations [2]. We and others discovered that G-CSF also acts as a growth factor in the brain, and shows protective and regenerative properties in a number of CNS disease models [3-6]. The mechanisms leading to these beneficial effects likely include a combination of anti-apoptotic activity on neurons [5], stimulation of neurogenesis [5], enhancement of vessel formation [3], mobilization of bone marrow derived cells [4] and systemic anti-inflammatory effects [5]. Recently we described that G-CSF was protective in a mouse model of amyotrophic lateral sclerosis (ALS), the major adult-onset motoneuron disease. G-CSF treatment increased survival and volume of spinal α motoneurons and protected motoneuronal cell lines (NSC34) in vitro against apoptotic stimuli [6]. Importantly, while adult motoneurons prominently express the receptor for G-CSF [6,7] our studies did not unequivocally show that G-CSF exerted protection in this ALS model through its neuroprotective activity.

Here, we sought to determine whether G-CSF was able to protect motoneurons in vivo in a model of neonatal sciatic nerve axotomy. This experimental paradigm has been largely described, is thought to rely almost exclusively on the apoptotic machinery [8], and provides a powerful tool to study the neuroprotective activity of growth factors towards motoneurons in vivo [4,5,9-13].

## Results

### The G-CSF receptor is expressed on neonatal motoneurons, and induced by axotomy

**Figure 1:**
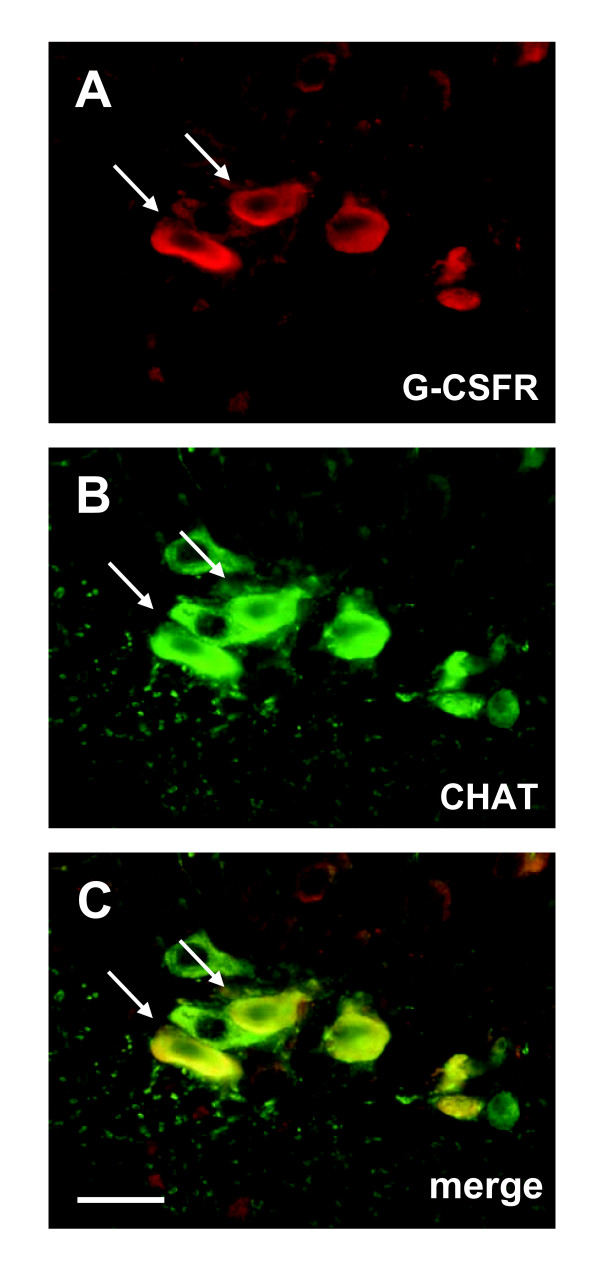
**The G-CSF receptor is expressed by motoneurons in the spinal cord of wt neonatal mice**. **(A-C) **Double fluorescence immunostaining of G-CSF receptor and CHAT (choline acetyltransferase) in the neonatal mouse spinal cord ventral horn. CHAT is used as a marker for motoneurons. All defined α motoneurons express G-CSFR (indicated by an arrow). All photomicrographs with 40× original magnification (OM), size bar 25 μm.

We have previously shown that the receptor of G-CSF (G-CSFR) is expressed on motoneurons in the adult spinal cord. We first determined whether the G-CSF receptor was expressed on motoneurons during early postnatal development. At postnatal day 9 (P9) we noted expression of the receptor on α motoneurons in the spinal cord ventral horn, identified by the following criteria: Choline-Acetyltransferase (CHAT) expression, an identifiable nucleolus, and a size larger than 300 μm^2 ^in the horizontal dimension (Figure 1). Invariably, all motoneurons defined by these criteria expressed the receptor.

**Figure 2:**
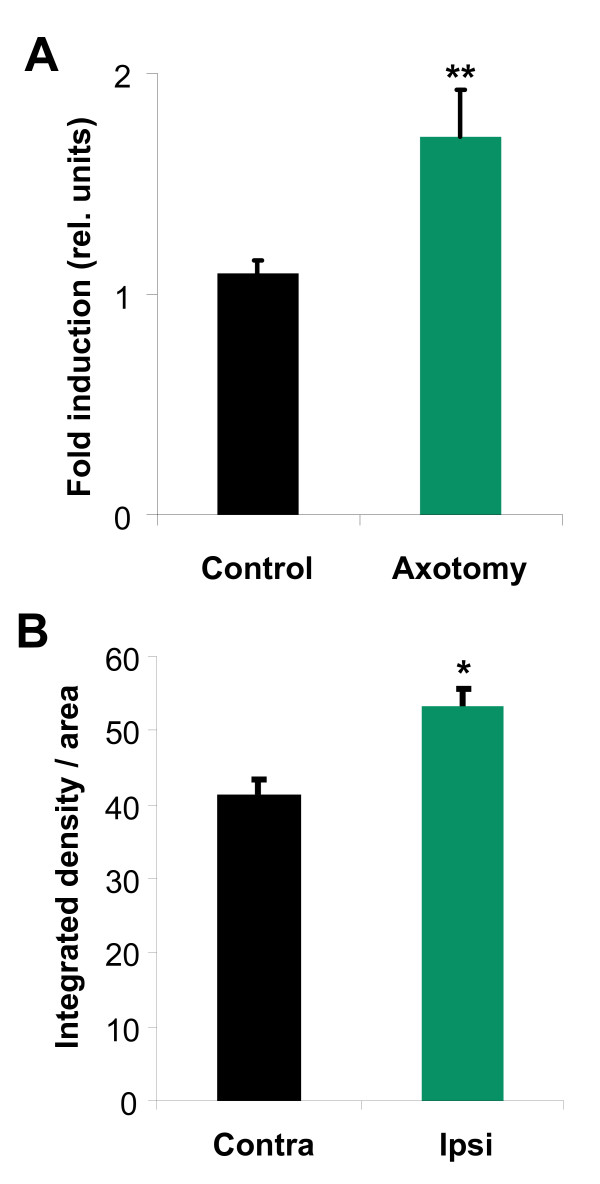
**G-CSF receptor expression after sciatic nerve axotomy**. (A) G-CSF receptor is upregulated after sciatic nerve axotomy in spinal cord of neonatal mice. Quantitative PCR for G-CSF receptor of spinal cords of axotomized or not neonatal mice (induction of 55%; n = 3, **p < 0.005). (B) G-CSF receptor staining intensity is stronger on the motoneurons after axotomy. Fluorescent intensity of G-CSF receptor staining on motoneurons was calculated on the ipsi- and contralateral side with an image processing program (*p < 0.05, n = 49).

The expression of G-CSF receptor is induced by neurons under neurodegenerative conditions such as after cerebral ischemia or in ALS, presumably as an endogenous protective response [6,7]. We therefore examined expression of the G-CSF receptor by quantitative PCR of whole spinal cords 4 days following neonatal axotomy of the right sciatic nerve (i.e. on postnatal day 9). Indeed, we found that G-CSFR expression in the whole spinal cord increased by 55% (p < 0.005) (Figure 2A).

To clarify whether this induction likely originated from the injured motoneurons, we quantified fluorescent staining intensities of ipsi- and contralateral motoneurons from the lumbar spinal cord region. We detected significantly stronger protein expression on ipsi- versus contralateral motoneurons, suggesting that axotomized motoneurons upregulate the receptor in response to injury (p < 0,05) (Figure 2B).

In conclusion, the G-CSF receptor is expressed at the neonatal stage and its expression is increased in response to axotomy.

### CNS- targeted overexpression of G-CSF protects motoneurons against cell death in neonatal pups

Neonatal motoneuron death following sciatic nerve axotomy constitutes a widely used and well- established model of apoptotic motoneuron death that has been used to examine protective effects of trophic factors.

We chose to use transgenic mice that overexpress G-CSF in the CNS as an endogenous "delivery system" to study effects of increased concentration of G-CSF on the lesioned motoneurons. We used transgenic mice harbouring a bidirectional tta-responsive construct with expression cassettes for murine G-CSF and EYFP (pBEG). These mice were crossed with mice expressing the Tet-transactivator under control of the CaMKII α;-promoter, similar to the approach used in Pitzer et al. except for the use of the Thy1-driver used therein [7]. In the following we compare three types of transgene combinations in littermates derived from crosses of mice heterozygous for the transgene CaMKII-tTA and heterozygous for pBEG. The three groups are a.) mice not harbouring any transgene ("wt"), b.) mice harbouring only the BEG2 transgene (which do not express G-CSF), and c.) mice harbouring both the BEG2 transgene and the CaMKII-tTA driver, that do express G-CSF.

**Figure 3:**
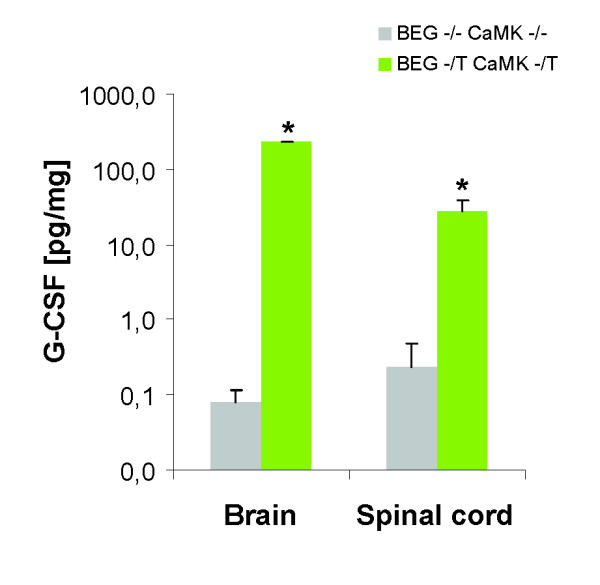
**G-CSF concentration in the CNS**. Levels of G-CSF are higher in the brain and in the spinal cord of mice overexpressing G-CSF (BEG -/T CaMK -/T) compared to wild type littermate controls (BEG -/- CaMK -/-). G-CSF proteins were quantified by ELISA with protein lysates from brain or spinal cord (n = 3, *p < 0.05).

Although expression via the CaMKII driver in our transgenes is mainly in the forebrain, release of G-CSF in the spinal cord is expected from long corticospinal axonal connections. Indeed, the CaMKIIα;-promoter directed G-CSF overexpression in the forebrain leads to a strong elevation of G-CSF levels in the neonatal brain and spinal cord (P9; 28.00 ± 11.60 vs 0.24 ± 0.23 pg/mg tissue protein; p < 0.05) (Figure 3).

**Figure 4:**
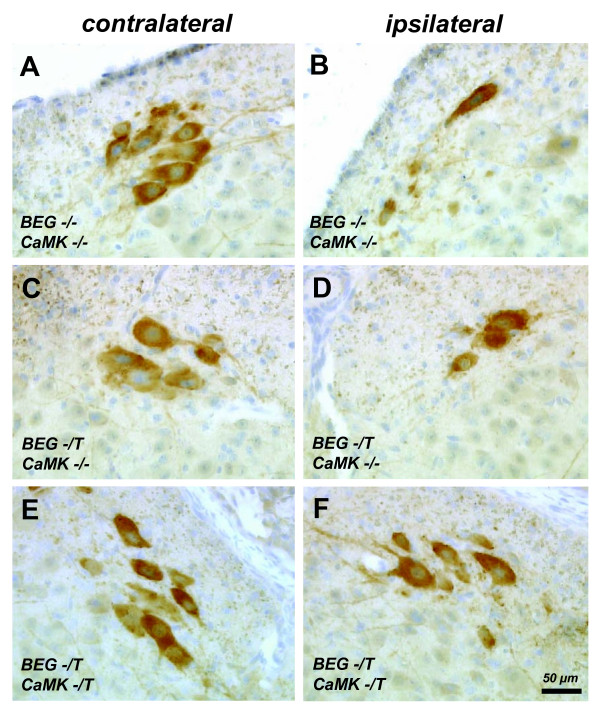
**Examples of the histological evaluation of CHAT positive cells in the ventral horn of the lumbar spinal cord of neonatal mice after sciatic nerve axotomy**. Sections from the lumbar spinal cord were stained with CHAT antibodies. (A, B) Examples of motoneurons in the ventral horn contralateral and ipsilateral to the lesion side in BEG -/- CaMK -/- mice (wild type littermates). (C, D) Examples of lumbar sections contra- and ipsilateral to the lesion from a BEG -/T CaMK -/- littermate (non-G-CSF expressing). (E, F) Examples of lumbar sections contra- and ipsilateral to the lesion from a G-CSF overexpressor (BEG -/T CaMK -/T mouse). (B, D, F) There are more CHAT-positive cells detectable 4 days after axotomy on the ipsilateral side in mice overexpressing G-CSF (BEG -/T CaMK -/T). All photomicrographs with 20× original magnification (OM), size bar 50 μM.

**Figure 5:**
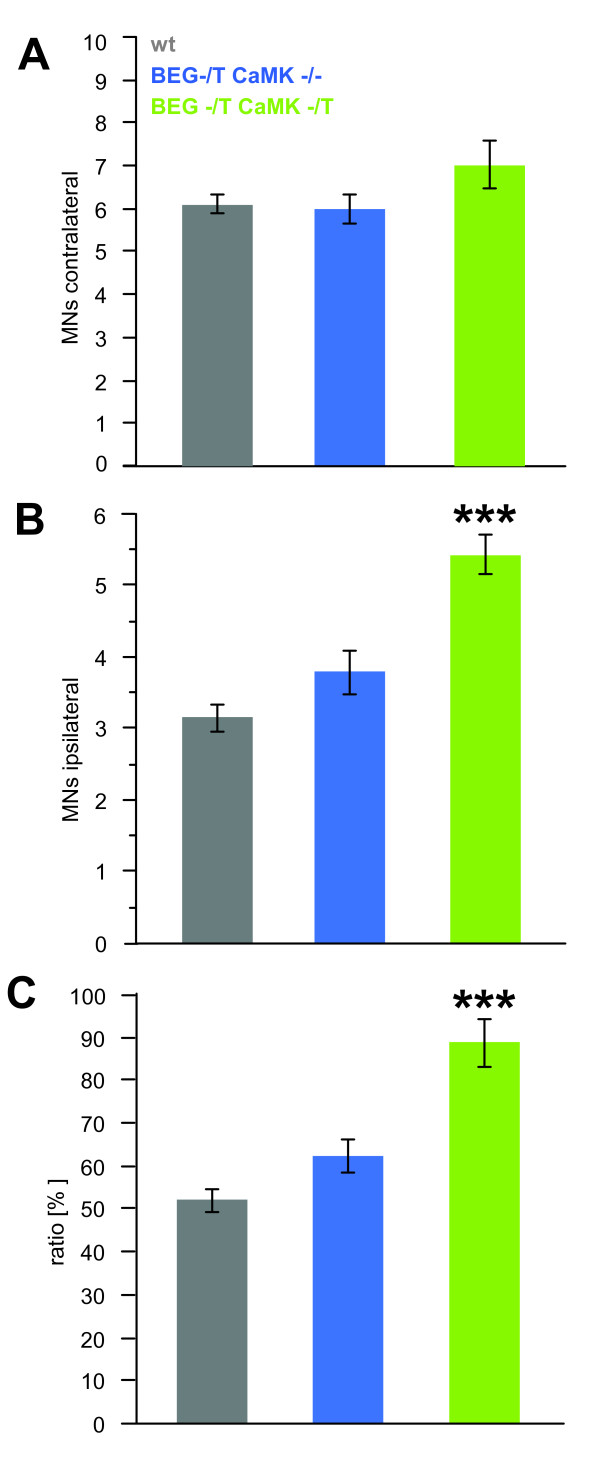
**Overexpression of G-CSF improves motoneuron survival following sciatic nerve axotomy in neonatal pups**. (A) Quantification of the absolute number of α; motoneurons per section of the lumbar spinal cord on the contralateral (unlesioned) side. Numbers are not significantly different between the three genotypes studied. (B) On the axotomized side numbers of detectable motoneurons are dramatically decreased in the littermate animals lacking the CaMK-tta driver transgene (wt, BEG -/- CaMK-/-; and BEG -/T CaMK-/-), whereas overexpression of G-CSF in the CNS results in significantly higher numbers of surviving motoneurons (***p < 0.0001 by ANOVA and Fisher's LSD post-hoc analysis). (C) Percentage of surviving motoneurons on the ipsilateral (axotomized) relative to the corresponding contralateral (control) side. While only 50 - 60% of the motoneurons survive in the genotypes lacking the CaMK-tta driver transgene, close to 90% of the motoneurons survive in the presence of the driver (i.e. in animals overexpressing G-CSF) (***p < 0.0001 by ANOVA and Fisher's LSD post-hoc analysis). (Number of animals: wt, n = 13; BEG -/T CaMK -/-, n = 6; BEG -/T CaMK -/T, n = 7; 6 sections were studied per animal).

Offspring from crosses of BEG and CaMK-tta transgenic mice were subjected to complete unilateral sciatic nerve axotomy at postnatal day 5 (P5). We analyzed effects on motoneuron survival in the lumbar section of the spinal cord (L4/5 levels) after 4 days at P9. Examples of the histological appearance of CHAT positive cells in the ventral horn of the lumbar spinal cord of neonatal mice after sciatic nerve axotomy are given in figure 4. Our axotomy model results in a loss of approximately 40 - 50% of motoneurons 4 days after the injury (Figure 4A and 4B; Figure 5A and 5B), a result consistent with other studies [8,9]. Already apparent from the histological sections was that clearly more motoneurons survive in mice with the G CSF transgene activated by the CaMK-tta driver (BEG -/T CaMK -/T) (compare figure 4B and 4F).

We then quantified numbers of motoneurons in the lumbar spinal cord of P9 neonatal mice contra- and ipsilateral to the side of axotomy. The α motoneurons were defined as (i) being situated in the ventral horn of the lumbar region; (ii) having a clearly identifiable nucleolus and staining positive for CHAT and (iii) having a minimum cross-sectional area of 300 μm^2^.

There was no difference between the absolute numbers of motoneurons on the contralateral side between the three genotypes used, although there was a non-significant trend for slightly higher numbers in the double-transgenic group (BEG -/T CaMK-/T (7.03 ± 0.39 motoneurons/section) vs. BEG -/T CaMK-/- (5.98 ± 0.38) and wt (6.1 ± 0.28); p = 0.1 by ANOVA) (Figure 5A). On the axotomized side, sciatic nerve axotomy dramatically decreased the numbers of motoneurons in animals lacking the CaMK-tta driver transgene, whereas double transgenic animals showed significantly higher numbers of surviving motoneurons (BEG -/T CaMK-/T: 5.43 ± 0.28 motoneurons/section; BEG -/T CaMK-/-: 3.79 ± 0.27 and wt: 3.15 ± 0.20; p < 0.0001 by ANOVA and Fisher's LSD post-hoc analysis) (Figure 5B). We also directly compared the fraction of surviving motoneurons relative to the unlesioned side (Figure 5C). This analysis revealed that only 50 - 60% of the motoneurons survived in the genotypes lacking the CaMK-tta driver transgene, whereas close to 90% of the motoneurons survived in the double transgenic mice (i.e. in animals overexpressing G-CSF) (BEG -/T CaMK-/T (88.76 ± 4.32%) vs. BEG -/T CaMK-/- (62.14 ± 4.22%) and wt (51.93 ± 3.02%); p < 0.0001 by ANOVA and Fisher's LSD post-hoc analysis).

### G-CSF preserves motoneuron size after axotomy

**Figure 6:**
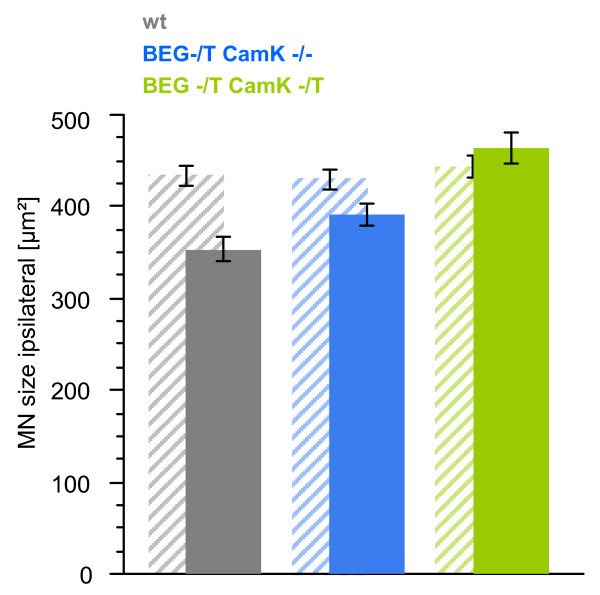
**Size distribution of surviving neurons in the spinal cord of neonatal mice following sciatic nerve axotomy**. On the contralateral side (hatched bars) the size distribution between the three genotypes is similar. On the ipsilateral side (filled bars) there is a downward shift in the mean size of the surviving motoneurons in the littermates without overexpression of G-CSF (BEG -/- CaMK -/-; BEG -/T CaMK -/-). (p < 0.0001 for BEG -/T CaMK -/T vs. BEG -/- CaMK -/-; **p = 0.0008 for BEG -/T CaMK -/T vs. BEG -/T CaMK -/-; ANOVA followed by Fisher's LSD; Number of motoneurons measured: wt, n = 301; BEG -/T CaMK -/-, n = 197; BEG -/T CaMK -/T, n = 227).

Our previous results in ALS mice had shown that the shrinking of motoneurons in the SOD1(G93A) transgenic mice was partially restored by G-CSF treatment. To determine whether this was also the case after sciatic nerve axotomy, we studied the distribution of motoneuron size of all motoneurons including those that are < 300 μm^2 ^determined by their cross-sectional area in the horizontal plane in all cells that were situated in the ventral horn, had a nucleolus, and were CHAT-positive. On the contralateral side, size distribution was similar between the three genotypes (Figure 6, hatched bars; BEG -/T CaMK -/T (442.55 ± 11.08 μm^2^); BEG -/T CaMK -/- (431.84 ± 12.52 μm^2^); wt (434.20 ± 9.74 μm^2^); p = 0.78 by ANOVA).

On the ipsilateral (axotomized) side, there was a downward shift in the mean size of the motoneurons in the littermates that did not overexpress G-CSF (wt and BEG -/T CaMK-/-). On the contrary, double transgenic animals (BEG -/T CaMK -/T) displayed a relatively preserved motoneuron size, suggesting a healthier state of those cells (Figure 6, solid bars; BEG -/T CaMK -/T (464 ± 17 μm^2^); BEG -/T CaMK -/- (391 ± 12 μm^2^); wt (353 ± 13 μm^2^); p < 0.001; ANOVA followed by Fisher's LSD). This result was also obtained if we only consider the α motoneurons that were counted for determining cell survival (> 300 μm^2^): 555.85 ± 17.43 μm^2 ^(BEG -/T CaMK -/T); 484.32 ± 19.73 μm^2 ^(BEG -/T CaMK -/-); 477.68 ± 17.93 μm^2 ^(wt); p = 0.0029 by ANOVA. Additional file 1 shows a histogram distribution of motoneurons contra- and ipsilateral to the axotomy. A detailed summary of all data measured is given in additional file 2.

## Discussion

Here we show that the receptor for G-CSF is expressed by neonatal motoneurons and that G-CSF protects lumbar motoneurons in a model of neonatal sciatic nerve axotomy. We have chosen this model because of the clarity of the involved pathophysiology to obtain more insight into the nature of G-CSF's effect on motoneurons. Sendtner et al. first described rescue of motoneuron death caused by neonatal axotomy by the growth factor CNTF [12]. This was followed by demonstration of a number of other neurotrophic factors protective in this paradigm. A series of papers then defined this axotomy-induced motoneuron death as apoptosis, with involvement of bcl proteins, AKT, IAPs, and caspases [14-16]. Overexpression of G-CSF protects lumbar motoneurons to an extent that is comparable to many of the classic neurotrophic factors. The experiments done here are therefore a further step in redefining G-CSF as a neurotrophic factor.

On top of a relative increase of motoneuron numbers following overexpression of G-CSF, we also noted an increase of about 100 μm^2 ^in the mean cross-sectional area of surviving motoneurons. This suggests a functionally more active, or healthier state of those motoneurons. This has also been noted in adult SOD1(G93A) mice by us previously [7]. This finding might point to an additional trophic effect of G-CSF in addition to counteracting apoptosis. Alternatively, this could signify a proportionally greater anti-apoptotic effect size of G-CSF on larger motoneurons which potentially express more receptors on the cell surface.

We have studied motoneuron survival 4 days after axotomy with the aim to demonstrate that G-CSF can counteract apoptosis in motoneurons in vivo in a simple paradigm as a mean to better dissect mechanisms of action in the more complex ALS mouse models. We do not know how transient this effect may be, and whether increased survival would still be detectable at 1 month after axotomy. All studies with growth factors in axotomy models that have examined varied time points after axotomy found that the number of surviving neurons declined with time to varying degrees in spite of ongoing treatment [17,18]. The "slope" of this decline might give an indication of the relative potency of growth factors in this paradigm (e.g [18]). However, there was always a lasting effect observed if growth factors showed protection after shorter observation time points. The purpose of our study was however not to compare relative efficacies of growth factors in this paradigm. We prefer to study questions of relative efficacy and effect stability in disease models closest and of relevance to the human indication targeted, which is ALS and SOD1-mutant mice in our case.

The observation that the G-CSF receptor was induced by axotomy parallels findings from other neurological disease models, such as stroke, spinal cord injury, or ALS [6,7], and underlines the notion that the G-CSF system is part of an endogenous protective system for neurons. This is certainly an argument for the use of G-CSF as a pharmacological agent that adresses an endogenously pre-specified mechanism. Indeed, G-CSF is currently under clinical investigation in ischemic stroke [19,20], and ALS [21-23].

We have undertaken this experiment to better dissect mechanisms responsible for the previously demonstrated beneficial function of G-CSF in the SOD1(G93A) mouse model for ALS. We have chosen the axotomy model to clearly answer the question whether G-CSF exerts a direct influence on motoneuron apoptosis in vivo. We have demonstrated potent anti-apoptotic properties of this "redefined" neurotrophic factor in vitro either in cortical neurons [6], or in motoneurons [7], and we favoured the anti-apoptotic hypothesis due to the strong effect on motoneuron survival in vivo. However, ALS is a multifaceted disease, with many factors involved in the pathophysiology [24-26], and the SOD1(G93A) models reproduce this complexity with discussed involvement of the neuromuscular junction, the immune system, glial cells and others. Moreover, G-CSF is a pleiotropic factor that for example also impacts on immunocompetence and inflammatory reactions. The data presented here clearly demonstrate a potent effect of G-CSF on motoneuron apoptosis in vivo, and support our assumption that anti-apoptosis plays a central role in the observed effects in the SOD1 transgenic ALS model.

Since G-CSF has such a potent protective role in apoptosis mediated by cell-inherent mechanisms as a response to nerve damage the interesting question arises whether the G-CSF system might also play a role in developmental death of motoneurons. This has indeed been shown for a number of neurotrophic factors that protect in the axotomy paradigm [27]. We do not believe that this is a confounding factor in our case since the expression of the CaMKII driven tTA is switched on during P2 and P5 [28-30], while the sensitive period for physiological motoneuron death is between E14 and P3 [27].

Although we do not see significant effects on the number of contralateral motoneurons in the different transgenic crosses, there is an interesting trend (p = 0.1) towards more motoneurons contralaterally in the G-CSF overexpressors compared to littermates not transgenic for the CaMKII tta driver. One might speculate that a reason for this trend observation could be contralateral effects of axotomy that are counteracted by G-CSF. This slight nominal difference on the contralateral side does however not change our result, since the protective effect of G-CSF is also highly significant when regarding ipsi-/contralateral ratios instead of ipsilateral cell numbers.

The mechanisms responsible for counteraction of apoptosis elicited by G-CSF in neurons have been previously delineated by us: Activation of AKT in neurons and induction of stat3 and bcl-proteins [6]. The AKT and stat3 pathways play a crucial role for the survival of the axotomized motoneurons [31,32]. It is therefore highly likely that G-CSF mediates its protective effect via these pathways in the axotomy paradigm as well.

## Conclusions

Here we have shown that G-CSF counteracts motoneuron death in a model of neonatal axotomy-induced apoptosis. This is most likely mediated by direct actions of the protein on motoneurons in vivo, and suggests that the protection offered by G-CSF in ALS mouse models is due to its direct neuroprotective activity. Moreover, our data strengthen the overall evidence that G-CSF is a candidate drug for the treatment of ALS.

## Methods

### Generation of G-CSF-overexpressing mice

Generation of the BEG transgenic lines is also described in [7]. We cloned the cDNA for murine G-CSF in a bi-directional Tet-transactivator (tta) responsive vector (pBI, Clontech). EYFP was inserted on the other side of the promoter for easy visualization of expression. To generate the pBI-EYFP-G-CSF ("pBEG") plasmid, G-CSF was amplified from a mouse brain cDNA library and inserted into the pBI Tet vector (Clontech) using the restriction sites NheI and EcoRV. EYFP was inserted into the multiple cloning site of the vector. Transgenic mice ("BEG") were generated, and selected for copy number integration. Copy numbers were estimated by quantitative PCR on genomic DNA by comparing pBEG/cyclophilin ratios of wild type and founder mice. For generating CNS- target G-CSF overexpressing mice, mice of line BEG6 were crossed with mice expressing the Tet-transactivator under control of the CaMKII-promoter (CaMKII-tta) [33], and successful activation of the construct was verified by EYFP imaging.

### Sciatic nerve injury

Neonatal mice were anesthetized and immobilized by hypothermia after placement on ice, and unilateral sciatic nerve section was performed on postnatal day 5 (P5). The skin of the right leg was incised parallel to the femur. To expose the sciatic nerve a longitudinal cut was made through the biceps femoris muscle with a sharpened forceps. The right sciatic nerve was transected at midthighlevel, a small piece (about 1-2 mm in length) of the nerve was removed to prevent reinnervation and the overlying skin of the thigh was sutured. Pups were warmed and returned to the mother. All animal manipulation followed current regulations of and were approved by the Regierungspräsidium Karlsruhe, Germany.

### Immunohistochemistry

After deep anesthesia spinal cords were removed rapidly, immersed in 4% PFA, and embedded in paraffin. For immunofluorescence, microtome sections of paraffin-embedded tissues (10 μm) were deparaffinated and microwaved (citrate buffer at 600 W for 15 min). For double-fluorescence staining, sections were incubated simultaneously with the G-CSF receptor antiserum (SC-694; Santa Cruz Biotechnology, Santa Cruz, CA, USA; 1:100) and the CHAT antibody (AB144P; Chemicon Europe Ltd., UK; 1:100) at 4°C overnight. After enhancing the G-CSF receptor staining by adding a biotinylated anti-rabbit secondary antibody (Dianova, Hamburg, Germany; 1:200), sections were incubated with a Streptavidin-coupled fluorophore (Invitrogen, Karlsruhe, Germany; 1:200) or the appropriate fluorescence-coupled secondary antibody (Dianova, Hamburg, Germany; 1:200). The nuclei were counterstained with Hoechst 33342 (Molecular Probes, 1:10000). Controls included omission of primary antibodies, fluorescence swapping and single-fluorescence stainings. Pictures were captured by video camera (Olympus DP71) coupled to a fluorescent microscope (Olympus IX80). To quantify staining intensity for the G-CSF receptor, pictures were analyzed with an image processing program (ImageJ). Intensity of grey levels were calculated for each motoneuron and normalized to its area.

For light-microscopic CHAT-staining coronal paraffin sections from the lumbar spinal cord (P9) were stained with the CHAT antibody using the avidin-biotin complex technique with DAB as chromogen (DakoCytomation), and counterstained with hemalaun.

### Counting of motoneurons

Counting of motoneurons was performed at postnatal day 9 (P9), i.e. 4 days following axotomy at P5. Spinal cord paraffin sections of a thickness of 10 μm, spaced by 100 μm over a length of 0.5 mm, were counted at the spinal level L4-L5. Motor axons that constitute the sciatic nerve in C57/bl6 mice originate from L3 to L5 [34]. All neurons in the ventral horn were counted as α motoneurons if they had a clearly identifiable nucleolus, were >300 μm^2 ^in size and were CHAT-positive. Counting was performed with pictures acquired with the LMD 6000 microscope (Leica) and its system program.

### Quantitative reverse transcription polymerase chain reaction

RNA was isolated from spinal cords using RNeasy Mini kit (Qiagen). Complementary DNA was synthesized from 1 mg total RNA using oligo-dT primers and superscript III reverse transcriptase (Invitrogen), according to standard protocols. Quantitative reverse transcription-polymerase chain reaction (RT-PCR) was performed using the Lightcycler system (Roche) with SYBR-Green staining of double-stranded DNA. Cycling conditions were as follows: 10 min at 95°C; 5 s at 95°C, 10 s at the annealing temperature of 64°C, 30 s at 72°C for 50 cycles and 10 min at 95°C. The following primers was used: 'G-CSFR-2582s':TGT GCC CCA ACC TCC AAA CCA;'G-CSFR-2817as':GCT AGG GGC CAG AGA CAG AGA CAC. Product specificity was ensured by melting curve analysis and electrophoresis on agarose gel. Relative regulation levels were derived after normalization to cyclophilin: 'cyc5':ACC CCA CCG TGT TCT TCG AC;'acyc300':CAT TTG CCA TGG ACA AGA TG.

### Statistics

Experiments were performed in a completely randomized and blinded manner. The experimenter was blinded to the genotype of the animals at the time of the experiment, and at the time of histological evaluation. For comparisons of 2 groups the ttest was employed. For 3 group comparisons, ANOVA was used, followed by post-hoc Fisher's test for contrasts. A p value < 0.05 was considered statistically significant. Statistical analyses were done using JMP 7.0.1 (SAS Institute).

## Authors' contributions

CP, LD, AH and AS conceived and planned the experiments, AS performed statistical analyses, AH conducted all experiments. CP, LD, AH and AS wrote the manuscript. All authors read and approved the final manuscript.

## Supplementary Material

Additional file 1 **Description: Shown is the histogram distribution of CHAT-positive cells in L4/5 for the 3 different genotypes examined, and for the ipsi- and contralateral side**. Click here for file

Additional file 2 **Description: Table of the motoneuron quantification data**. Given are the absolute numbers and percentages of all CHAT positive cells; the mean size of all CHAT positive cells and the number of a motoneurons per section for the 3 genotypes examined (each for the ipsilateral and contralateral side). Click here for file
