# Supplementary figures and images for: G-CSF protects motoneurons against axotomy-induced apoptotic death in neonatal mice
Source: BMC Neurosci. 2010 Feb 23;11:25. doi: 10.1186/1471-2202-11-25 (PMC2844381; doi:10.1186/1471-2202-11-25)

***ipsilateral***

**BEG -/T CaMK-/-**

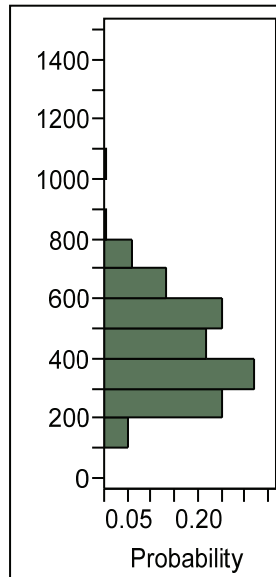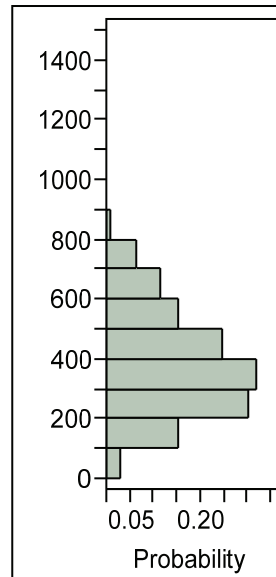

**BEG -/T CaMK-/T**

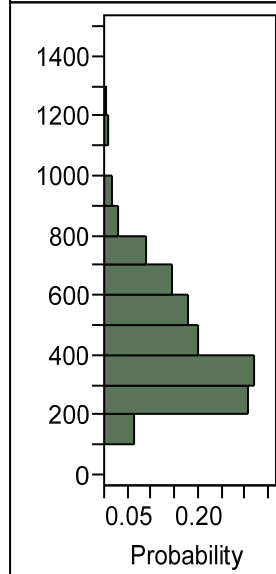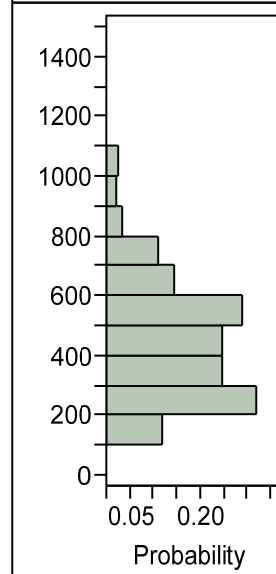

**BEG -/- CaMK-/-**

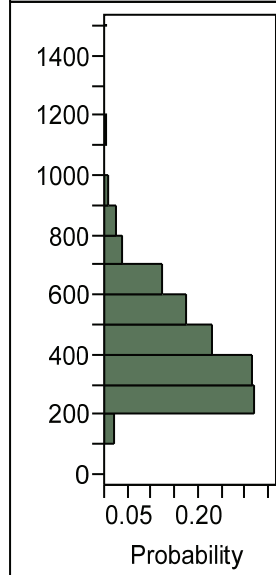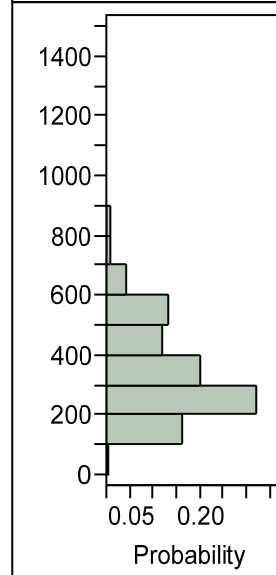

Supplement: Additional file 1 — Description: Shown is the histogram distribution of CHAT-positive cells in L4/5 for the 3 different genotypes examined, and for the ipsi- and contralateral side. [file 1471-2202-11-25-S1.pdf]
