# Supplementary material for: G-CSF protects motoneurons against axotomy-induced apoptotic death in neonatal mice
Source: BMC Neurosci. 2010 Feb 23;11:25. doi: 10.1186/1471-2202-11-25 (PMC2844381; doi:10.1186/1471-2202-11-25)
Supplement: Additional file 2 — Description: Table of the motoneuron quantification data. Given are the absolute numbers and percentages of all CHAT positive cells; the mean size of all CHAT positive cells and the number of a motoneurons per section for the 3 genotypes examined (each for the ipsilateral and contralateral side). [file 1471-2202-11-25-S2.pdf]

Additional file 2: Motoneuron quantification data.

	BEG -/- CaMK -/-		***BEG -/T CaMK -/-***		***BEG -/T CaMK -/T***		***p value (ANOVA)***
	contralateral						
	N	***%***	***N***	***%***	***N***	***%***	
000-100 µm²	0	0	0	0	0	0	
100-200 µm²	6	2	9	4	14	5	
200-300 µm²	98	25	46	19	68	23	
300-400 µm²	97	25	58	25	70	23	
400-500 µm²	71	18	40	17	44	15	
500-600 µm²	54	14	46	19	39	13	
600-700 µm²	38	10	24	10	32	11	
700-800 µm²	12	3	11	5	20	7	
>800 µm²	14	3	2	0	14	4	
N counted (all CHAT+)	390		236		301		
N counted (>300 µm²)	286		181		219		
Mean size all CHAT+ cells [µm²]	**434±10**		**432±13**		**443±11**		*n.s.*
Mean size α motoneurons (>300 µm²) [µm²]	**502±10**		**491±13**		**520±12**		*n.s.*
α motoneurons / section	**6.1±0.28**		**5.98±0.38**		**7.03±0.39**		*n.s.*
	ipsilateral						
	N	***%***	***N***	***%***	***N***	***%***	
000-100 µm²	1	0	4	2	0	0	
100-200 µm²	49	16	22	11	17	7	
200-300 µm²	96	32	43	22	46	20	
300-400 µm²	61	20	45	23	36	16	
400-500 µm²	36	12	35	18	36	16	
500-600 µm²	40	13	22	11	42	19	
600-700 µm²	13	4	16	8	21	9	
700-800 µm²	2	1	9	5	16	7	
>800 µm²	2	1	1	1	12	5	
N counted (all CHAT+)	301		197		227		
N counted (>300 µm²)	155		128		164		
Mean size all CHAT+ cells [µm²]	**353±13**		**391±12**		**464±17**		*0.0008*
Mean size α motoneurons (>300 µm²) [µm²]	**478±18**		**484±20**		**556±17**		*0.0029*
α motoneurons / section	**3.15±0.20**		**3.79±0.27**		**5.43±0.28**		*<0.0001*
